# Long-Term Auditory, Tinnitus, and Psychological Outcomes After Cochlear Implantation in Single-Sided Deafness: A Two-Year Prospective Study

**DOI:** 10.3390/jcm15020644

**Published:** 2026-01-13

**Authors:** Jasper Karl Friedrich Schrader, Moritz Gröschel, Agnieszka J. Szczepek, Heidi Olze

**Affiliations:** 1 Department of Otorhinolaryngology, Head and Neck Surgery, Charité—Universitätsmedizin Berlin, Corporate Member of Freie Universität Berlin and Humboldt Universität zu Berlin, 10117 Berlin, Germany; jasper-karl-friedrich.schrader@charite.de (J.K.F.S.); moritz.groeschel@charite.de (M.G.); agnes.szczepek@charite.de (A.J.S.); 2 Faculty of Medicine and Health Sciences, University of Zielona Gora, 65-046 Zielona Gora, Poland

**Keywords:** cochlear implantation, single-sided deafness, patient-reported outcomes, tinnitus, quality of life, spatial hearing, stress and anxiety

## Abstract

**Background/Objectives**: Single-sided deafness (SSD) impairs speech perception, reduces spatial hearing, decreases quality of life, and is frequently accompanied by tinnitus. Cochlear implantation (CI) has become an established treatment option, but long-term prospective evidence across multiple functional and psychological domains remains limited. This study investigated auditory performance, subjective hearing outcomes, tinnitus burden, and psychological well-being over a two-year follow-up in a large SSD cohort. **Methods**: Seventy adults with SSD underwent unilateral CI. Assessments were conducted preoperatively and at 6 months, 1 year, and 2 years postoperatively. Outcome measures included the Freiburg Monosyllable Test (FS), Oldenburg Inventory (OI), Nijmegen Cochlear Implant Questionnaire (NCIQ), Tinnitus Questionnaire (TQ), Perceived Stress Questionnaire (PSQ), Generalized Anxiety Disorder scale (GAD-7), and General Depression Scale (ADS-L). Longitudinal changes were analyzed using Wilcoxon signed-rank tests with effect sizes; Holm-adjusted *p*-values were applied for baseline-to-follow-up comparisons. **Results**: Speech perception improved markedly within the first 6 months and remained stable through 2 years, with large effect sizes. All OI subdomains demonstrated early and sustained improvements in subjective hearing ability. Several hearing-related quality-of-life domains assessed by the NCIQ, particularly social interaction, self-esteem, and activity participation, showed medium-to-large long-term improvements. Tinnitus severity decreased substantially, with marked reductions observed by 6 months and maintained thereafter; the proportion of tinnitus-free patients increased at follow-up, although tinnitus symptoms persisted in a substantial subset of participants. Perceived stress was reduced initially at the early follow-up and remained below baseline thereafter. Anxiety and depressive symptoms mostly stayed within nonclinical ranges, showing no lasting changes after adjusting for multiple comparisons. **Conclusions**: In this prospective cohort, cochlear implantation was associated with durable improvements in auditory outcomes, tinnitus burden, and selected patient-reported quality-of-life domains over two years. Although significant functional and patient-centered improvements were noted, persistent tinnitus and diverse psychosocial outcomes underscore the need for personalized counseling and comprehensive follow-up that incorporate patient-reported outcomes and psychological assessments.

## 1. Introduction

Hearing loss is one of the most common communication disorders worldwide and may affect one or both ears. When severe-to-profound sensorineural hearing loss occurs in only one ear while hearing remains normal in the other, the condition is classified as single-sided deafness (SSD). SSD is defined by a severe-to-profound hearing loss (≥70 dB HL pure-tone average across 0.5, 1, 2, and 4 kHz) in the impaired ear, together with normal to mildly impaired hearing (≤30 dB HL) in the better-hearing ear [1]. According to the German S2k guideline of the German Society of Oto-Rhino-Laryngology, Head and Neck Surgery, SSD additionally requires ≤60% monosyllable recognition with a hearing aid in the Freiburg test at 65 dB SPL [2,3].

SSD can be prelingual (present at birth or early in development) or postlingual (occurring after language acquisition) [4]. Clinically, postlingual SSD often results from sudden sensorineural hearing loss [5], Ménière’s disease [6], vestibular schwannoma and its treatments [7], temporal bone fractures [8], and infections [9,10]. In some cases, the cause remains unknown (idiopathic).

Patients with SSD face a unique mix of disabling auditory and psychosocial challenges. Unilateral severe-to-profound hearing loss (i.e., loss of binaural hearing due to unilateral deafness) results in poor speech understanding in noise, diminished spatial hearing and sound localization, and increased listening effort, often leading to fatigue and communication difficulties. Sounds from the deaf side are weakened by head shadow, reducing auditory awareness and hindering participation in everyday listening situations. Additionally, tinnitus is prevalent and often the primary symptom of SSD [11,12]. Tinnitus frequently accompanies increased stress, anxiety, and depression [13]. Furthermore, with an average age of around 50 years, SSD patients are often still of working age [14,15,16,17], highlighting the occupational and social impacts of the condition. Treatment priorities for SSD patients are unique: in addition to recovering bilateral auditory cues, reducing tinnitus-related distress and psychological distress are key focuses [17,18,19]. Conversely, rehabilitation of individuals with asymmetric hearing loss (AHL) or bilateral (double-sided) deafness (DSD) usually focuses on restoring communication, independence, and social engagement [20,21,22]. These differences emphasize the importance of clearly distinguishing SSD, AHL, and DSD populations in both clinical practice and research. Overall, these sensory and psychological issues can limit social participation, damage self-confidence, and negatively affect quality of life, underlining the importance of effective long-term rehabilitation methods.

Treatment options for SSD include Contralateral Routing of Signal (CROS) hearing aids, bone-anchored hearing systems (BAHS), and cochlear implants (CI). Cochlear implantation is a well-established and effective treatment option for SSD [11,14,15,16,17,18,23,24,25]. Compared to CROS or BAHS, CI provides greater improvements in speech perception, quality of life, and tinnitus burden—even in cases of unilateral deafness [14,15,16,26]. Notably, CI is the only intervention capable of restoring true binaural hearing by actively reintroducing auditory input to the deafened ear. This mechanism can re-enable sound localization and improve spatial awareness [25].

Despite the increasing clinical use of CI for SSD, the broader effects of cochlear implantation—beyond audiological outcomes—have rarely been systematically examined [27]. Long-term follow-up data for patients with SSD are also limited, and existing longitudinal studies often focus solely on speech perception, bilateral hearing, and tinnitus outcomes [14,27,28,29]. Although several studies have examined the auditory- and tinnitus-related effects of cochlear implantation in SSD, long-term evidence remains scarce, with available longitudinal research primarily focusing on speech perception, spatial hearing, and tinnitus. The few studies with follow-up periods of 2 years or more—such as the long-term cohorts by Mertens and colleagues—assessed binaural hearing, tinnitus distress, and hyperacusis but did not include broader psychological outcomes, such as perceived stress, anxiety, or depression [30,31,32]. However, none of the previous long-term studies used disease-specific measures such as the Nijmegen Cochlear Implant Questionnaire (NCIQ), leaving the long-term effects of cochlear implantation on quality of life in SSD largely unexamined. Furthermore, none of these long-term studies used a comprehensive patient-reported outcome (PRO) framework that includes general psychological health. Consequently, the long-term course of psychological well-being following cochlear implantation in SSD remains mostly unknown. This gap underscores the need for prolonged follow-up studies that assess not only auditory and tinnitus-related changes but also the broader psychological effects of cochlear implantation on daily life and emotional wellness.

Building on this growing evidence base, preliminary analyses from our cohort (*n* = 20) have already demonstrated short-term benefits [17,18,19]. These studies showed additional benefits of CI, including tinnitus relief, reduced perceived stress, and improved disease-specific quality of life in patients with SSD and AHL. However, these earlier analyses lacked long-term follow-up and did not include a thorough patient-reported outcome framework that evaluates psychological factors such as stress, anxiety, or depression.

To address this gap, the present prospective single-center study investigated the long-term effects of cochlear implantation in a well-defined group of adults with SSD over two years using a standardized PRO battery. Specifically, this study explored (1) the clinical benefits that SSD patients achieve two years after CI surgery and (2) the timing of these improvements and whether they remain stable over time.

## 2. Materials and Methods

### 2.1. Patient Cohort

This prospective study included 70 adult patients (28 men, 42 women) with unilateral postlingual deafness who received a CI between 2013 and 2023 at the Department of Otolaryngology at Charité–Universitätsmedizin Berlin. The local Ethics Committee approved data collection (standing permit EA2/030/13). All participants signed informed consent forms.

#### Sample Size Considerations

This study was designed as a longitudinal observational cohort of all adults with single-sided deafness who received a cochlear implant at our tertiary referral center during the predefined recruitment period and consented to a 2-year follow-up. The sample size of 70 participants, therefore, reflects a consecutive, real-world cohort rather than a formally fixed target. Previous longitudinal studies on cochlear implantation in single-sided deafness have typically included much smaller cohorts (often ≤30 participants) and rarely extended follow-up beyond 2 years. Against this background, our cohort is comparable to or larger than most existing longitudinal SSD–CI samples and thus provides a robust basis for estimating trajectories of patient-reported outcomes over time. From a statistical perspective, a sample of 70 participants provides adequate power to detect within-subject changes of small to moderate magnitude in our primary patient-reported outcomes. Under standard assumptions (α = 0.05, two-sided), this sample size allows detection of standardized effect sizes of approximately 0.3–0.4 for paired comparisons between time points, which is in the range considered clinically relevant for changes in subjective hearing and psychological measures. The study was therefore primarily powered to detect clinically meaningful longitudinal changes rather than small effects in subgroup analyses.

**Inclusion Criteria**

Participants were eligible for inclusion if they met all of the following criteria:

- Age ≥ 18 years at the time of implantation.
- Postlingual single-sided deafness (SSD) as defined by:
  a. Severe-to-profound sensorineural hearing loss in the poorer ear (pure-tone average ≥70 dB HL at 0.5, 1, 2, and 4 kHz), and
  b. Normal hearing or maximally mild hearing loss in the contralateral ear (air-conduction threshold ≤30 dB HL).
- Speech recognition score ≤ 60% in the poorer ear with a hearing aid, as assessed using the Freiburg monosyllable test at 65 dB SPL (per German S2k guideline).
- Intact speech perception in the better ear, defined as ≥80% in the Freiburg monosyllable test at 70 dB SPL.
- Scheduled for unilateral cochlear implantation performed at the study center (Charité–Universitätsmedizin Berlin).
- Ability and willingness to complete the full patient-reported outcomes (PRO) test battery.
- Written informed consent to participate in the study.

**Exclusion Criteria**

Participants were excluded if any of the following applied:

- Age < 18 years.
- Prelingual deafness or congenital unilateral hearing loss.
- Follow-up period < 6 months, or insufficient postoperative data for PRO analysis.
- Relevant language barriers preventing reliable completion of questionnaires.
- Presence of neurological, cognitive, or psychiatric conditions that could impair valid participation or questionnaire responses.
- Previous cochlear implantation or other active middle/inner ear interventions affecting the poorer ear.
- Active middle ear pathology, contraindicating CI surgery at the time of evaluation.
- Bilateral hearing loss or asymmetric hearing loss (AHL; defined below) not meeting criteria for SSD.

Baseline data collection included patient age, duration of deafness, pure-tone and speech audiometry, and standardized patient-reported outcome (PRO) measures preoperatively and at 6 months, 1 year, and 2 years postoperatively. Asymmetric hearing loss was defined as clinically relevant interaural asymmetry in hearing thresholds (e.g., PTA4 difference ≥30 dB) and impaired hearing in the better ear; therefore, these cases did not meet the SSD definition used in this study.

### 2.2. Audiometric Tests

#### The Freiburg Monosyllable Test

The Freiburg Monosyllable Test (Freiburger Einsilbertest, FS) is among the most commonly used clinical speech audiometry tools in German-speaking countries [33]. It aims to evaluate speech intelligibility at the word level without relying on contextual or semantic cues. The test involves lists of 20 phonetically balanced monosyllabic words, presented at a specified sound pressure level—typically 65 dB SPL for normal conversational volume or 70 dB SPL in some guidelines. Because the words are presented in isolation and lack linguistic context, the test offers a precise and sensitive measure of auditory resolution, especially under challenging conditions such as single-sided deafness.

Performance is expressed as a percentage of correctly repeated words (0–100%). Lower scores reflect poorer speech recognition and greater functional hearing impairment. In patients with SSD, the Freiburg Monosyllable Test is useful for evaluating the auditory contribution of the deafened ear, as the contralateral ear is either masked or excluded, depending on the clinical protocol.

In this study, speech intelligibility with a hearing aid on the ear scheduled for CI was evaluated preoperatively using the Freiburg Monosyllable Test at 65 dB in free-field conditions, with a loudspeaker positioned at 0°. The opposite ear was masked with white noise. The Freiburg Monosyllable Test was repeated under similar conditions and using the same settings postoperatively, with unilateral CI and contralateral masking, at 3 time points (6 months, 1 year, 2 years).

### 2.3. Patient-Reported Outcomes Test Battery

A comprehensive set of validated PROs from the Charité test battery was administered at 4 time points (preoperative, 6 months, 1 year, 2 years). The following tests were used:

#### 2.3.1. Oldenburg Inventory (OI)

The Oldenburg Inventory is a standardized self-report measure of subjective hearing ability in everyday listening situations [34,35]. It includes three subscales that assess speech understanding in quiet, speech understanding in noise, and directional hearing. Items are rated on a 5-point Likert scale from 1 (“very poor”) to 5 (“very good”), with higher scores indicating better perceived hearing performance. Subscale scores and a total score are calculated as the average of the items.

#### 2.3.2. Nijmegen Cochlear Implant Questionnaire (NCIQ)

The NCIQ is a disease-specific instrument that assesses health-related quality of life among cochlear implant users [36]. It comprises 60 items across six subdomains: basic sound perception, advanced sound perception, speech production, self-esteem, activity limitations, and social interactions. Responses are given on a 5-point Likert scale and converted into scores from 0 (poor) to 100 (excellent). Higher scores indicate better functioning and quality of life in the respective areas.

#### 2.3.3. Tinnitus Questionnaire (TQ)

The Tinnitus Questionnaire measures the severity and impact of tinnitus-related distress [37]. It comprises 52 items addressing emotional and cognitive distress, intrusiveness, auditory-perceptual difficulties, sleep problems, and physical complaints. Items are scored on a 3-point scale, yielding a total score ranging from 0 to 84. Severity levels are categorized as mild, moderate, severe, or very severe. Scores ≥ 47 suggest decompensated (unhabituated) tinnitus that requires clinical attention.

#### 2.3.4. Perceived Stress Questionnaire (PSQ)

The PSQ is a validated 20-item tool that assesses perceived stress over the past four weeks [38,39,40]. It evaluates four components: worries, tension, joy (reverse scored), and demands. Items are rated on a 4-point Likert scale, and responses are normalized to a total score ranging from 0 to 1. Higher scores signify greater perceived stress. Values between 0.45 and 0.60 indicate moderate stress, while values above 0.60 indicate high stress.

#### 2.3.5. General Depression Scale (ADS-L)

The ADS-L is a 20-item screening tool for depressive symptoms based on the Center for Epidemiologic Studies Depression Scale (CES-D) [41,42]. Items cover affective, cognitive, and somatic aspects of depression and are rated on a 4-point frequency scale. Total scores range from 0 to 60, with higher values indicating greater symptom burden. A cutoff of ≥23 points is used to identify clinically relevant depressive symptoms.

#### 2.3.6. Generalized Anxiety Disorder-7 (GAD-7)

The GAD-7 is a validated 7-item questionnaire used to screen for symptoms of generalized anxiety over the past two weeks [43]. Each item is scored on a 4-point Likert scale (0–3), yielding a total score of 0–21. Higher scores reflect greater anxiety severity. Established thresholds categorize symptom severity as minimal (0–4), mild (5–9), moderate (10–14), and severe (15–21). A score ≥ 10 serves as the clinical cutoff for clinically significant anxiety, warranting further evaluation.

### 2.4. Statistical Methods

Statistical analyses were performed using IBM SPSS Statistics (version 30.0, IBM Corp., Armonk, NY, USA). The normality assumptions were not met for several variables; therefore, nonparametric methods were employed. Descriptive statistics are reported as median and interquartile range (Q1–Q3) for patient-reported outcomes and as mean ± standard deviation for audiometric measures, unless stated otherwise.

Longitudinal changes across the four time points (preoperative/baseline, 6 months, 1 year, and 2 years) were assessed using Friedman tests when complete repeated measurements were available. For planned pairwise contrasts, Wilcoxon signed-rank tests were used to compare baseline with each follow-up time point (baseline vs. 6 months, baseline vs. 1 year, baseline vs. 2 years). Pairwise comparisons between follow-up visits were considered exploratory and are reported descriptively. Changes in tinnitus prevalence (tinnitus present, defined as TQ Total > 0, vs. absent, defined as TQ Total = 0) were analyzed using McNemar’s exact test for paired binary data (baseline vs. 6 months, 1 year, and 2 years), with Holm step-down adjustment applied to these three prevalence comparisons.

Multiplicity control: To reduce the risk of Type I error, Holm correction was applied within each outcome across the three baseline-to-follow-up comparisons (6 months, 1 year, 2 years). Holm-adjusted *p*-values are provided in Supplementary Table S1.

Effect sizes and confidence intervals: For Wilcoxon signed-rank tests, effect sizes were expressed as the matched-pairs rank-biserial correlation (r_rb_), calculated from the sums of positive and negative ranks as r_rb_ = ((W+) − (W−))/((W+) + (W−)) [44]. For each comparison, the number of available paired observations (n [pairs]) and the number of non-zero, paired differences (n [non-zero diffs]) are reported. Ninety-five percent confidence intervals for r_rb_ were obtained using nonparametric bootstrap resampling of paired observations (2000 resamples; participants resampled with replacement while preserving within-subject pairing) [45] and are reported in Supplementary Table S1. Effect-size magnitude was interpreted using conventional benchmarks (0.1 = small, 0.3 = medium, 0.5 = large) [46].

Missing data: To assess potential attrition bias, baseline characteristics were compared between participants with complete 2-year core outcome data (“completers”) and those without complete 2-year data (“non-completers”) (Supplementary Table S2). Continuous variables are presented as median [Q1–Q3] and compared using Mann–Whitney U tests. Categorical variables are presented as *n* (%) and compared using Fisher’s exact test. All plots were created with biorender.com.

## 3. Results

### 3.1. Demographic and Clinical Characteristics

The patients’ baseline characteristics are presented in Table 1.

Follow-up completeness varied by outcome due to missing assessments at later time points; therefore, sample sizes differed across analyses. Baseline comparisons between participants with available versus missing 2-year follow-up data showed no substantial differences in key baseline characteristics and core outcomes (Supplementary Table S2).

### 3.2. Audiometric Outcomes

**Figure 1:**
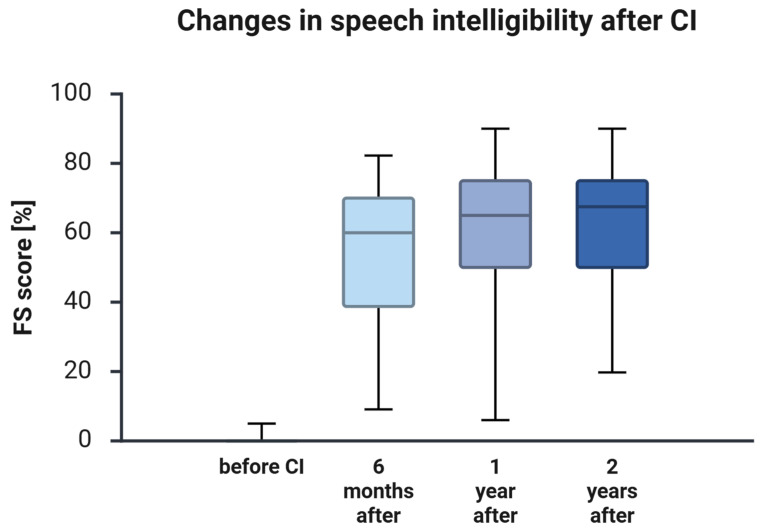
Speech intelligibility improves in patients with SSD following CI. Boxplots show Freiburg monosyllable speech recognition scores (FS, %) measured preoperatively (baseline) and at 6 months, 1 year, and 2 years after cochlear implantation in patients with SSD. The horizontal line indicates the median, boxes represent the interquartile range (Q1–Q3), and whiskers indicate the range. Inferential statistics for baseline-to-follow-up comparisons (Holm-adjusted *p*-values within outcome) and effect sizes with 95% confidence intervals are provided in Supplementary Table S1.

Before cochlear implantation, speech recognition in the implanted (poorer) ear was essentially absent, with a mean Freiburg monosyllable score of 1.15% ± 4.78% (Figure 1). Scores increased markedly by 6 months to 53.46% ± 23.59% and remained high at 1 year (60.70% ± 23.82%) and 2 years (61.60% ± 21.50%). Improvements relative to baseline were statistically significant after Holm correction (see Supplementary Table S1; baseline vs. 6 months: Z = −5.972, *p* < 0.001; baseline vs. 2 years: Z = −4.945, *p* < 0.001). Changes between follow-up visits were comparatively small: speech understanding improved modestly from 6 months to 1 year (Z = −2.457, unadjusted *p* = 0.014) and remained stable from 1 to 2 years (Z = −1.649, unadjusted *p* = 0.099). Overall, speech understanding showed pronounced early gains within the first 6 months, followed by sustained performance through 2 years.

### 3.3. Changes in PRO Subjective Hearing Assessment (OI)

**Figure 2:**
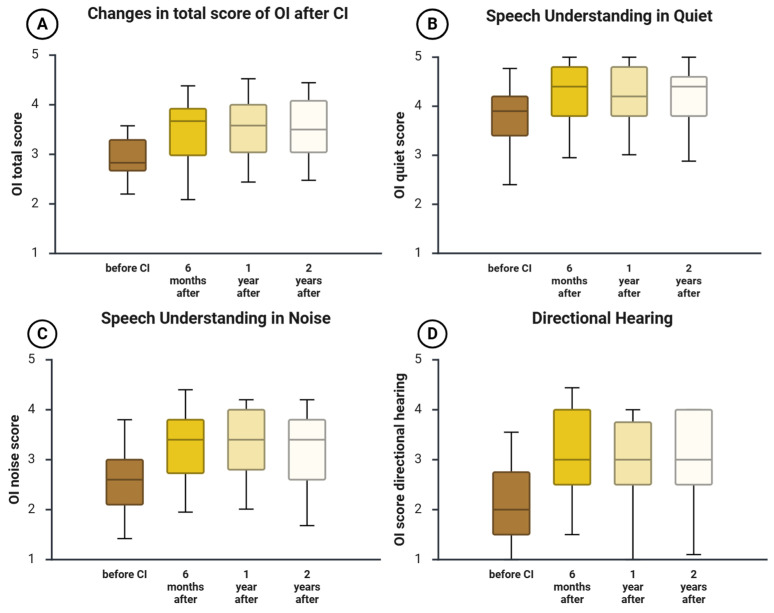
Changes in hearing ability were subjectively assessed using the Oldenburg Inventory at 6 months, 1 year, and 2 years after CI. Boxplots depict OI scores at baseline, 6 months, 1 year, and 2 years after cochlear implantation: (**A**) OI total score, (**B**) speech understanding in quiet, (**C**) speech understanding in noise, and (**D**) directional hearing. The horizontal line indicates the median, boxes represent the interquartile range (Q1–Q3), and whiskers indicate the range. Inferential statistics for baseline-to-follow-up comparisons (Holm-adjusted *p*-values within outcome) and effect sizes with 95% confidence intervals are provided in Supplementary Table S1.

Subjective hearing performance in daily life was evaluated using the Oldenburg Inventory (OI), which captures clinically relevant aspects of speech understanding in quiet, speech understanding in noise, and directional hearing. Changes over time were examined to characterize the pattern and stability of patient-perceived hearing benefits following cochlear implantation (Figure 2). For baseline-to-follow-up comparisons, Holm-adjusted *p*-values and effect sizes with confidence intervals are provided in Supplementary Table S1.

#### 3.3.1. Speech Understanding in Quiet

At baseline, the median score was 3.9 (25th–75th percentile: 3.4–4.2). Scores increased by 6 months (median 4.4, 3.8–4.8) and remained similar at 1 year (4.2, 3.8–4.8) and 2 years (4.4, 3.8–4.6). Improvements relative to baseline were statistically significant after Holm correction at all follow-ups (Holm-adjusted *p* ≤ 0.001), indicating a sustained gain in subjective speech understanding in quiet.

#### 3.3.2. Speech Understanding in Noise

Baseline performance in noise was limited, with a median of 2.6 (2.15–3.0). Scores improved by 6 months (3.4, 2.73–3.8) and remained stable at 1 year (3.4, 2.8–4.0) and 2 years (3.4, 2.6–3.8). All improvements relative to baseline were statistically significant after Holm correction (Holm-adjusted *p* < 0.001), supporting a robust and lasting benefit in noise.

#### 3.3.3. Directional Hearing

At baseline, the median directional hearing score was 2.0 (1.5–3.0). Scores increased by 6 months (3.0, 2.38–4.0) and remained stable at 1 year (3.0, 2.5–4.0) and 2 years (3.0, 2.5–4.0). Improvements relative to baseline were statistically significant after Holm correction at all follow-ups (Holm-adjusted *p* < 0.001), indicating sustained improvement in perceived sound localization.

#### 3.3.4. Total OI Score

The total OI score improved from a baseline median of 3.04 (2.67–3.38) to 3.70 (3.15–4.11) at 6 months, with scores remaining similar at 1 year (3.75, 3.11–4.08) and 2 years (3.75, 3.17–4.17). Improvements relative to baseline were statistically significant after Holm correction at all follow-ups (Holm-adjusted *p* < 0.001), reflecting a substantial and durable overall improvement in subjective hearing ability after implantation.

### 3.4. Changes in the PRO Health-Related Quality of Life

#### 3.4.1. NCIQ1—Basic Sound Perception

At baseline, the median score for basic sound perception was 70 (25th–75th percentile: 57.5–80.62). Scores increased modestly at 6 months (73.75, 62.5–83.12), 1 year (75, 59–86.5), and 2 years (75, 60.56–87.5). None of the baseline-to-follow-up comparisons remained statistically significant after Holm correction (Holm-adjusted *p* = 0.126 at 6 months and 1 year; 0.062 at 2 years; Supplementary Table S1), indicating a slow, long-term trend rather than a robust early change.

#### 3.4.2. NCIQ2—Advanced Sound Perception

Advanced sound perception showed minimal change over time. The baseline median was 72.5 (64.38–85) and remained similar at 6 months (77.5, 64.82–87.5), 1 year (77.78, 65–87.5), and 2 years (77.5, 63.12–85). No baseline-to-follow-up comparison was statistically significant after Holm correction (Holm-adjusted *p* = 0.427 for all baseline contrasts; Supplementary Table S1), indicating stable advanced sound perception over two years.

#### 3.4.3. NCIQ3—Speech Production

Speech production remained stable across all time points. The baseline median was 87.5 (67.5–95.56), with comparable values at 6 months (86.11, 69.44–92.86), 1 year (86.11, 71.81–95), and 2 years (83.33, 72.22–94.69). No baseline-to-follow-up comparison was statistically significant after Holm correction (Holm-adjusted *p* = 0.823 at 6 months and 1 year; 0.467 at 2 years; Supplementary Table S1), consistent with high baseline speech-production scores and no measurable CI-related change in this domain.

#### 3.4.4. NCIQ4—Self-Esteem

Self-esteem improved over time. The baseline median was 53.9 (43.92–62.5), increasing to 58.33 (43.44–67.99) at 6 months and 57.5 (47.35–70) at 1 year, with further improvement by 2 years (57.5, 51.25–72.36). Improvements relative to baseline were statistically significant after Holm correction at all follow-ups (Holm-adjusted *p* = 0.008 at 6 months; 0.003 at 1 year; 0.000739 at 2 years; Supplementary Table S1), indicating sustained psychosocial benefit over time.

#### 3.4.5. NCIQ5—Activity Limitations

Activity limitations improved early and remained higher through 2 years. The baseline median was 52.5 (39.73–64.17), increasing to 57.5 (46.74–72.5) at 6 months, 58.33 (43.75–81.88) at 1 year, and 60 (45–75) at 2 years. Improvements relative to baseline were statistically significant after Holm correction at all follow-ups (Holm-adjusted *p* = 0.001 at 6 months; 0.000499 at 1 year and 2 years; Supplementary Table S1), supporting a durable increase in activity-related functioning after implantation.

#### 3.4.6. NCIQ6—Social Interaction

Social interaction showed the most consistent improvement. The baseline median was 55.9 (43.75–65.89), increasing to 61.11 (50–75.7) at 6 months, 67.5 (44.44–78.12) at 1 year, and 67.86 (53.89–75) at 2 years. Improvements relative to baseline were statistically significant after Holm correction at all follow-ups (Holm-adjusted *p* = 0.000325 at 6 months; 3.85 × 10^−5^ at 1 year; 6.51 × 10^−5^ at 2 years; Supplementary Table S1), indicating a robust and lasting enhancement in social functioning over two years.

#### 3.4.7. NCIQ Total Score

**Figure 3:**
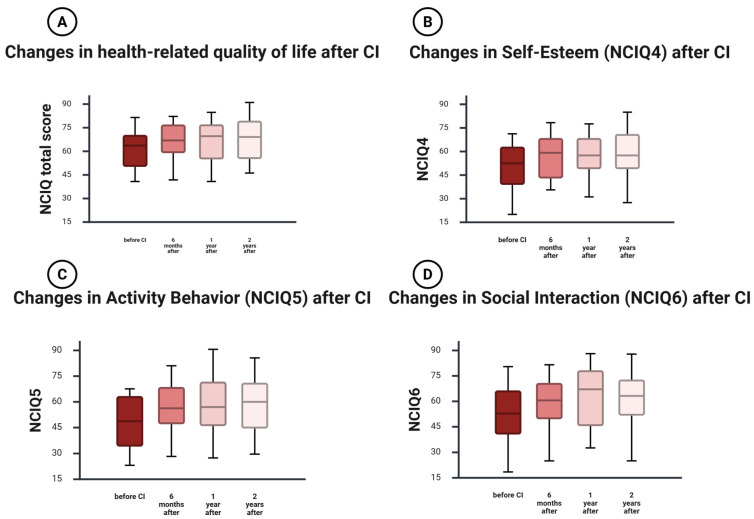
Changes in the health-related quality of life after CI. Boxplots show Nijmegen Cochlear Implant Questionnaire (NCIQ) domain scores and total score at baseline, 6 months, 1 year, and 2 years after cochlear implantation: (**A**) NCIQ total score, (**B**) self-esteem (NCIQ4), (**C**) activity limitations (NCIQ5), and (**D**) social interaction (NCIQ6). The horizontal line indicates the median, boxes represent the interquartile range (Q1–Q3), and whiskers indicate the range. Inferential statistics for baseline-to-follow-up comparisons (Holm-adjusted *p*-values within outcome) and effect sizes with 95% confidence intervals are provided in Supplementary Table S1.

The total NCIQ score increased over time, from a baseline median of 65.07 (55.78–72.65) to 67.57 (59.25–76.85) at 6 months, 69.74 (55.39–82.05) at 1 year, and 70.04 (56.4–79.13) at 2 years. Improvements relative to baseline were statistically significant after Holm correction at all follow-ups (Holm-adjusted *p* = 0.001), indicating sustained gains in health-related quality of life (Figure 3).

### 3.5. Tinnitus Prevalence

At baseline, tinnitus was reported by 93.4% of participants (tinnitus-free: 6.6%) based on available data. At follow-up, tinnitus prevalence was 80.0% at 6 months (tinnitus-free: 20.0%), 75.9% at 1 year (tinnitus-free: 24.1%), and 81.3% at 2 years (tinnitus-free: 18.8%). Thus, while the proportion of tinnitus-free patients increased after cochlear implantation, tinnitus symptoms persisted in a substantial subset of participants at all follow-up time points.

To statistically evaluate changes in tinnitus prevalence over time, paired categorical analyses were performed using McNemar’s exact test (baseline vs. each follow-up). In the overall sample with paired data, tinnitus prevalence was lower at 1 year compared with baseline after Holm correction (*p*_Holm = 0.0117). In contrast, the changes at 6 months and 2 years were not statistically significant after correction (both *p*_Holm = 0.0781). These patterns are visualized in Supplementary Figure S1.

Because the proportion of tinnitus-free participants decreased slightly from 24.1% at 1 year to 18.8% at 2 years, we additionally examined tinnitus prevalence in the subgroup with complete tinnitus data across all four time points (baseline, 6 months, 1 year, and 2 years; *n* = 41). In this full-dataset subgroup, tinnitus prevalence was 95.1% at baseline (tinnitus-free: 4.9%) and remained lower at follow-up (82.9% at 6 months; 78.0% at 1 year; 82.9% at 2 years), corresponding to tinnitus-free proportions of 17.1%, 22.0%, and 17.1%, respectively. McNemar’s exact test within this complete subgroup similarly showed a statistically significant reduction at 1 year after Holm correction (*p*_Holm = 0.0469), but not at 6 months or 2 years (both *p*_Holm = 0.250). Together, these findings suggest that the small 1- to 2-year change in tinnitus-free proportions reflects individual fluctuation and sampling variation rather than uniform loss of benefit.

### 3.6. Change in PRO Tinnitus Severity (TQ)

**Figure 4:**
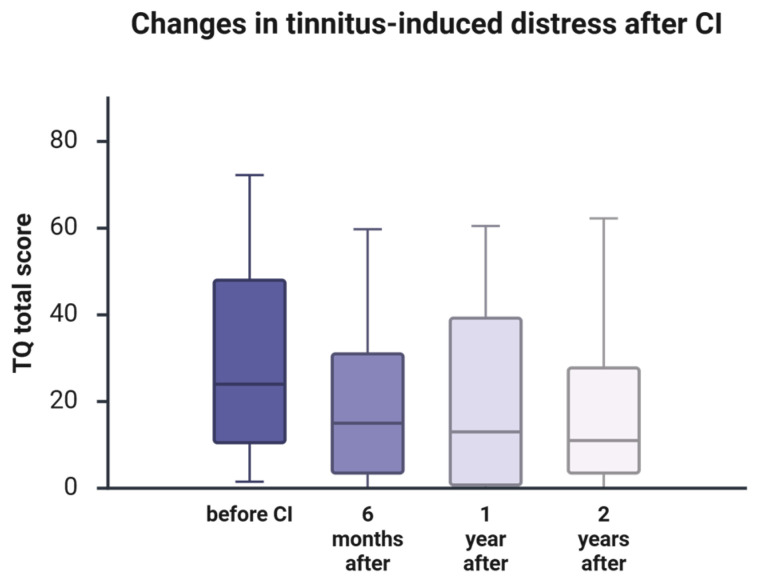
Changes in the TQ total score indicating tinnitus-related distress after CI. Boxplots show the Tinnitus Questionnaire total score (TQ total) at baseline, 6 months, 1 year, and 2 years post-cochlear implantation. The horizontal line indicates the median, boxes represent the interquartile range (Q1–Q3), and whiskers indicate the range. Inferential statistics for baseline-to-follow-up comparisons (Holm-adjusted *p*-values within outcome) and effect sizes with 95% confidence intervals are provided in Supplementary Table S1.

Tinnitus severity analyses were restricted to participants who reported tinnitus at baseline. Tinnitus-related distress decreased markedly after cochlear implantation, as reflected by the Tinnitus Questionnaire (TQ) total score (Figure 4). TQ scores showed a pronounced early improvement by 6 months, with sustained benefit thereafter; baseline-to-follow-up contrasts remained statistically significant after Holm correction, and effect sizes (with 95% CIs) supported the clinical relevance of these changes (Supplementary Table S1).

Before implantation, tinnitus distress was substantial and highly variable (median TQ 24; 25th–75th percentile: 11–51). By 6 months post-CI, the median TQ score had decreased to 9.5 (1.75–28), representing a statistically significant reduction after Holm correction (Holm-adjusted *p* < 0.001; Supplementary Table S1).

At 1 year, the median TQ score remained low at 10 (0.25–36.5), indicating maintenance of early improvement. No difference between follow-up visits (6 months vs. 1 year; Z = −0.835, unadjusted *p* = 0.404) was statistically significant, supporting stabilization after the initial postoperative reduction. Relative to baseline, the improvement at 1 year remained statistically significant after Holm correction, with a large effect size (Supplementary Table S1).

At 2 years, the median TQ score increased slightly but remained low at 11.5 (3.75–30.75). Again, no change was observed between 1 and 2 years (Z = −1.050, unadjusted *p* = 0.294). In contrast, the baseline-to-2-year improvement remained statistically significant after Holm correction, with a large effect size (Supplementary Table S1), indicating a stable and substantial long-term reduction in tinnitus-related distress.

### 3.7. Changes in PRO Stress Levels (PSQ)

**Figure 5:**
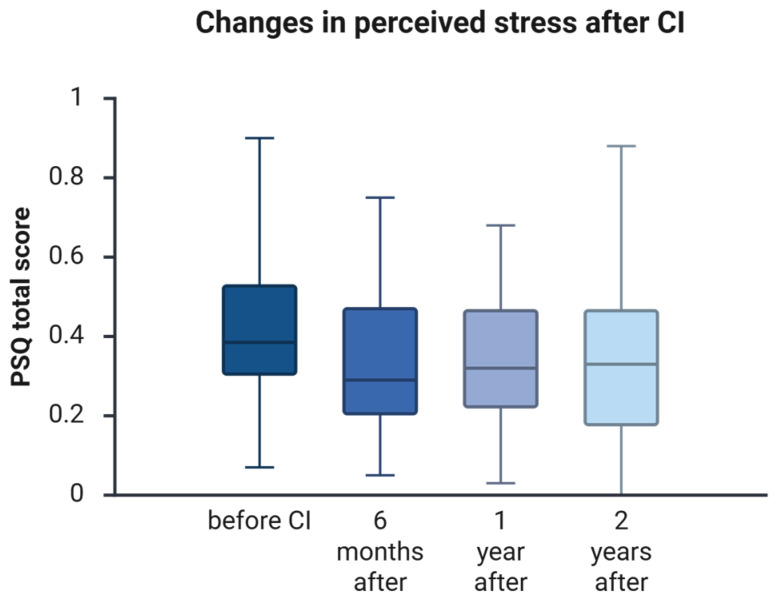
Changes in perceived stress after cochlear implantation. Boxplots show PSQ total scores at baseline (before CI) and at 6 months, 1 year, and 2 years after implantation. The center line indicates the median; boxes represent the interquartile range (25th–75th percentiles); whiskers indicate the minimum and maximum values. Lower PSQ total scores reflect lower perceived stress. Inferential statistics for baseline-to-follow-up comparisons (Holm-adjusted *p*-values within outcome) and effect sizes with 95% confidence intervals are provided in Supplementary Table S1.

Perceived stress was assessed using the PSQ total score (Figure 5). At baseline, the median PSQ score was 0.35 (25th–75th percentile: 0.23–0.52). By 6 months, scores decreased to 0.29 (0.18–0.47) and remained low at 1 year (0.28, 0.15–0.45), with a slight increase by 2 years (0.33, 0.18–0.47). All baseline-to-follow-up reductions were statistically significant after Holm correction (Holm-adjusted *p* = 0.001 at 6 months; 0.028 at 1 year; 0.037 at 2 years; Supplementary Table S1), indicating a sustained overall reduction in perceived stress relative to preoperative levels. No meaningful changes were observed between follow-up visits (6 months vs. 1 year: unadjusted *p* = 0.732; 1 year vs. 2 years: unadjusted *p* = 0.565), suggesting that the early improvement was maintained over time.

### 3.8. Changes in PRO Anxiety (GAD-7)

Anxiety symptoms were assessed with the GAD-7 (Supplementary Figure S2). At baseline, the median GAD-7 score was 4 (25th–75th percentile: 2–8). Scores remained low over time, with medians of 4 (1–7) at 6 months, 4 (1–6) at 1 year, and 5 (2–7) at 2 years. No baseline-to-follow-up comparison was statistically significant after Holm correction (Holm-adjusted *p* = 0.085 at 6 months; 0.174 at 1 year; 0.479 at 2 years; Supplementary Table S1). The baseline-to-6-month contrast showed a nominal decrease (unadjusted *p* = 0.028) that did not remain significant after adjustment, indicating overall stability of anxiety symptoms over the 2-year follow-up.

### 3.9. Changes in PRO Depressive Symptoms (ADS-L)

Depressive symptoms were assessed using the ADS-L (Supplementary Figure S2). At baseline, the median ADS-L score was 12 (25th–75th percentile: 7–23). Scores showed only minor fluctuations over time, with medians of 11 (5–20) at 6 months, 12.5 (6–20.75) at 1 year, and 11 (6–22) at 2 years. No baseline-to-follow-up comparisons were statistically significant after Holm correction (Holm-adjusted *p* = 0.273 at 6 months; 0.532 at 1 year; 0.532 at 2 years; Supplementary Table S1), indicating stable depressive symptom scores over the observation period.

## 4. Discussion

This prospective cohort study evaluated auditory outcomes, tinnitus burden, health-related quality of life, and psychological screening measures up to 2 years after cochlear implantation in adults with single-sided deafness. Across domains, the largest within-patient changes occurred within the first 6 months. Outcomes were generally stable from 1 to 2 years, supporting the durability of the observed benefits. This study explored two key questions about cochlear implantation in adults with SSD: (1) the level of patient benefits two years post-implantation, and (2) how these benefits evolve, including when early improvements occur and how stable they are. With a relatively large sample and assessments at 6 months, 1 year, and 2 years, we observed significant gains in auditory, tinnitus-related, psychosocial, and functional domains. Most improvements were observed within the first six months and remained stable over two years, indicating both a rapid initial response and long-term benefit. Overall, these results demonstrate that cochlear implants offer meaningful, lasting advantages for patients with SSD at two years, with a pattern of rapid early improvement followed by sustained stability.

### 4.1. Restoration of Bilateral Hearing

Unilateral hearing loss is a defining feature of SSD, causing significant challenges in speech-in-noise understanding. In this study, cochlear implantation quickly restored functional hearing in the implanted ear, with speech perception increasing from nearly zero preoperatively to over 50% at six months and remaining stable afterward. Subjective outcomes measured with the Oldenburg Inventory reflected these results, with particularly notable improvements in speech-in-noise perception and sound localization—the areas most affected by SSD.

Beyond these expected improvements, patients also reported better speech understanding in quiet, although baseline deficits in this domain are typically modest. Similar observations in other studies [15,17,19,23] suggest that CI-mediated restoration of bilateral hearing may enhance overall listening ease, even in conditions where monaural hearing would usually suffice. This underlines the functional relevance of reestablishing accurate bilateral auditory input [25].

### 4.2. Tinnitus Relief as a Major Therapeutic Effect

Tinnitus is a significant source of burden for patients with SSD, with prevalence estimates often exceeding 70–80% in pre-implantation cohorts [24,47,48]. In addition to reduced tinnitus-related distress, tinnitus prevalence declined after cochlear implantation, although symptoms persisted in a substantial subset of patients. Specifically, baseline tinnitus prevalence was 93.4% (6.6% tinnitus-free) and decreased to 75.9–81.3% across follow-ups (i.e., 18.8–24.1% tinnitus-free, depending on time point). The proportion free of tinnitus peaked at 1 year (24.1%). It was 18.8% at 2 years, which likely reflects fluctuations in tinnitus status and variation in available follow-up data rather than a uniform loss of benefit.

These findings align with previous research showing that CI benefits extend beyond auditory rehabilitation to include substantial and long-lasting tinnitus suppression [19,22,49,50]. Wendrich et al. also showed that cochlear implants (CI) provide better tinnitus suppression than bone-conduction implants. [49]. Potential mechanisms involve peripheral reafferentation and central auditory reorganization, initiated by stimulation of the previously inactive cochlear pathway [18,51]. Overall, the pattern of reduced distress, together with only partial resolution of tinnitus symptoms, highlights individual differences in treatment response and supports incorporating tinnitus-specific counseling and follow-up into routine SSD CI care.

### 4.3. Quality-of-Life Improvements: Participation, Self-Esteem, and Psychosocial Functioning

SSD can substantially limit daily participation, communication confidence, and social interactions. In this study, the NCIQ showed pronounced improvements in self-esteem, social engagement, and activity levels, with these changes emerging within 6 months and persisting for 2 years. These findings emphasize the broader everyday value of restored auditory input, enabling patients to re-engage socially, communicate more effectively, and experience reduced listening effort.

A detailed examination of NCIQ subscales revealed that improvements in auditory recognition of complex signals—such as music or speech via telephone—were less pronounced. This is consistent with previous studies indicating that benefits may be perceived as suboptimal in patients with SSD compared with those with bilateral hearing loss (DSD) [36,51,52,53], likely due to the preserved function of the contralateral ear.

Of particular interest is the sustained improvement in self-esteem (NCIQ4), which increased significantly at six months and continued to improve between years one and two. These findings suggest longer-term psychosocial adaptation processes and progressive integration of the implant into everyday life.

Despite significant gains, NCIQ subscale values 4–6 did not reach those of healthy controls at two years [36], indicating that subtle limitations in health-related quality of life may persist, potentially driven by residual tinnitus or challenges in complex listening environments.

### 4.4. Psychological Outcomes: Reduced Perceived Stress with Stable Anxiety and Depression Scores

Although baseline levels of anxiety and depressive symptoms in this cohort were below clinical thresholds, perceived stress was slightly elevated compared with general population reference values (~0.33) [39,40] (baseline median PSQ 0.35 [0.23–0.52]) [39,40], consistent with prior reports that SSD patients often experience higher stress than those with DSD or AHL [17,22]. Stress levels decreased significantly by six months postoperatively. They remained stable over the two years, suggesting a meaningful reduction in everyday strain following restoration of bilateral hearing and a decrease in tinnitus burden.

Anxiety and depressive symptoms showed some early postoperative improvements, although these were not consistently sustained over time and remained within non-clinical ranges. Variability may reflect attrition bias. Nonetheless, the overall pattern—improved stress, stable emotional health, and no deterioration in psychological measures—supports the emotional safety and secondary psychological benefits of cochlear implantation.

### 4.5. Alignment with SSD-Specific Core Outcomes (CROSSSD)

The outcome battery used in this study closely reflects the domains emphasized in the CORE Rehabilitation Outcome Set for SSD (CROSSSD) [54], namely activity limitations, participation restrictions, and self-reported hearing function. Our assessments of speech-in-noise performance, sound localization, tinnitus burden, participation-related quality of life, and psychological health cover all three domains. The consistently substantial improvements observed thus demonstrate that cochlear implantation provides benefits precisely where they matter most for SSD rehabilitation.

### 4.6. Summary and Clinical Relevance

Our findings show that cochlear implantation offers broad, rapid, and enduring benefits for adults with SSD. Improvements occur early—typically by six months—and remain stable for at least two years. These include measurable gains in speech-in-noise perception, spatial hearing, tinnitus relief, participation, psychosocial functioning, and perceived stress, all of which SSD has the most significant impact on. The large cohort and extended follow-up enhance the clinical significance of these results.

These outcomes underscore the importance of individualized preoperative counseling. Clinicians should emphasize that:

- most benefits occur within the first six months,
- improvements generally remain stable long-term,
- tinnitus relief is highly likely,
- psychosocial well-being may continue to improve even after the first year.

Tailored rehabilitation efforts should address both functional auditory challenges and psychosocial recovery.

### 4.7. Study Limitations

This study has several limitations. First, it is a single-center prospective cohort study, which may limit generalizability to other clinical settings and rehabilitation pathways. Additionally, the absence of a control group (e.g., CROS/BAHS, or no-treatment) precludes causal attribution and conclusions about comparative effectiveness. Therefore, results should be interpreted as associations within a longitudinal cohort rather than definitive evidence of superiority over alternative interventions.

Moreover, follow-up completeness decreased over time, which may introduce attrition bias. To mitigate this concern, we performed an attrition analysis comparing baseline characteristics and core outcomes between participants with and without available 2-year follow-up data (Supplementary Table S2). Nevertheless, unmeasured differences may persist. Although tinnitus distress decreased at the group level, tinnitus symptoms persisted in a substantial proportion of participants at follow-up, with clinical implications for preoperative counseling and post-implant management. Similarly, psychosocial screening measures showed heterogeneous and, in some domains, modest changes, indicating that improvements in auditory function and tinnitus may not uniformly translate into broader psychological benefits.

### 4.8. Outlook and Future Directions

Future research should include multicenter trials with larger and more diverse samples to enhance external validity and support standardized SSD management protocols. Comparative studies—ideally randomized or with matched control groups—are needed to evaluate how CI outcomes compare with those of CROS or BAHS devices, particularly in tinnitus suppression, spatial hearing, and quality-of-life domains.

Long-term follow-up beyond two years will be essential to elucidate the durability and evolution of auditory and psychosocial outcomes, including patterns of device use, effects of auditory training, and long-term adaptation. These efforts will refine patient selection, optimize counseling, and ultimately improve long-term rehabilitation outcomes for individuals with SSD.

## 5. Conclusions

Cochlear implantation in SSD was associated with durable improvements in auditory outcomes and tinnitus burden over two years, along with additional gains in several patient-reported quality-of-life domains. Patients experienced notable improvements in speech perception, subjective hearing ability, tinnitus burden, hearing-related quality of life, and perceived stress, mainly within the first six months. These improvements generally remained stable afterward. Psychosocial domains—particularly self-esteem, social interaction, and activity participation—improved over the long term. In contrast, anxiety and depressive symptoms showed no clinically meaningful deterioration and largely remained within nonclinical ranges. Taken together, these results suggest that cochlear implantation can provide sustained functional and patient-centered benefits in SSD; however, given the single-center design and the absence of a control group, the findings should be interpreted as longitudinal associations. The data further underscore the value of integrating patient-reported outcomes and psychological screening into routine follow-up and preoperative counseling to support expectation management and identify patients who may benefit from additional tinnitus- or psychosocially targeted interventions.

## Figures and Tables

**Table 1:** Baseline characteristics of the patient cohort.

Variable	Value at Baseline
Number of patients	70
Sex, *n* (%)	Male: 28 (40%); Female: 42 (60%)
Age at implantation (years)	Mean ± SD: 58.74 ± 13.30
Duration of deafness (months)	Mean ± SD: 13.06 ± 18.20

## Data Availability

Data are available from the corresponding author upon reasonable request.

## Supplementary Materials

The following supporting information can be downloaded at: https://www.mdpi.com/article/10.3390/jcm15020644/s1, Figure S1. Tinnitus prevalence over time; Figure S2. Anxiety and depressive symptoms over time; Table S1. Longitudinal changes from baseline to follow-up with effect sizes and 95% confidence intervals; Table S2. Attrition analysis: baseline characteristics of participants with versus without complete 2-year core outcome data.

## Abbreviations

The following abbreviations are used in this manuscript:

ADS-L	General Depression Scale (long version questionnaire)
AHL	Asymmetric Hearing Loss
CI	Cochlear Implant
DSD	Double-Sided Deafness (bilateral deafness)
FS	Freiburg Monosyllable Test (speech Intelligibility test)
GAD-7	Generalized Anxiety Disorder 7
NCIQ	Nijmegen Cochlear Implant Questionnaire (health-related quality of life)
OI	Oldenburg Inventory
PSQ	Perceived Stress Questionnaire
PRO	Patient Reported Outcome
SSD	Single-Sided Deafness (unilateral deafness)
TQ	Tinnitus Questionnaire
