# Supplementary material for: Long-Term Auditory, Tinnitus, and Psychological Outcomes After Cochlear Implantation in Single-Sided Deafness: A Two-Year Prospective Study
Source: J Clin Med. 2026 Jan 13;15(2):644. doi: 10.3390/jcm15020644 (PMC12842105; doi:10.3390/jcm15020644)
Supplement: Supplementary file 1 [file jcm-15-00644-s001.zip › Supplementary Figure S1.pdf]

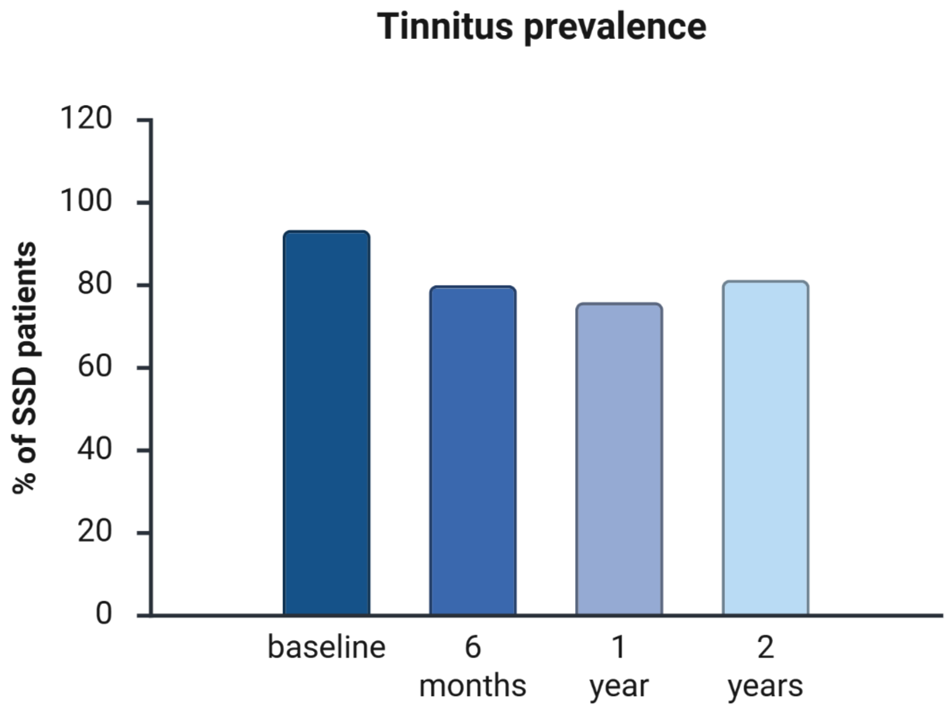

Supplementary Figure S1. Tinnitus prevalence over time.
Bar chart showing the proportion of participants reporting tinnitus at baseline and at 6 months, 1 year, and 2 years after cochlear implantation. Bars display tinnitus prevalence (%) at each time point; sample sizes (n) are shown above the bars. Tinnitus presence was defined as TQ Total > 0.
