# Supplementary material for: Long-Term Auditory, Tinnitus, and Psychological Outcomes After Cochlear Implantation in Single-Sided Deafness: A Two-Year Prospective Study
Source: J Clin Med. 2026 Jan 13;15(2):644. doi: 10.3390/jcm15020644 (PMC12842105; doi:10.3390/jcm15020644)
Supplement: Supplementary file 1 [file jcm-15-00644-s001.zip › Supplementary Figure S2.pdf]

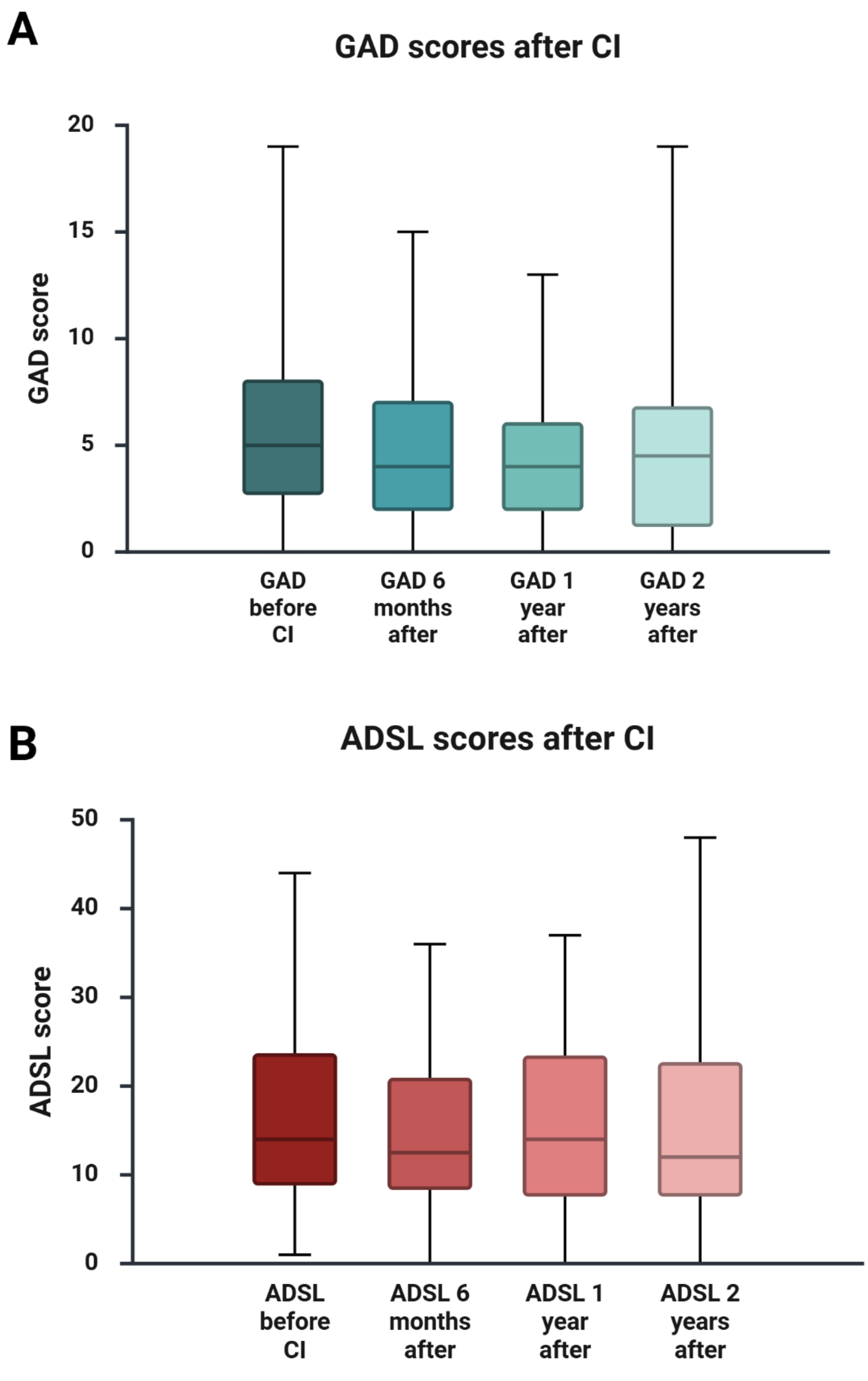

Supplementary Figure S2. Anxiety and depressive symptoms over time.
Boxplots show GAD-7 total scores (Panel A) and ADS-L total scores (Panel B) at baseline (before CI) and at 6 months, 1 year, and 2 years after implantation. The center line indicates the median; boxes represent the interquartile range (25th–75th percentiles); whiskers indicate min-max range. Higher scores indicate greater symptom burden; across follow-up, scores remained within nonclinical ranges.
