# Supplementary material for: Long-Term Auditory, Tinnitus, and Psychological Outcomes After Cochlear Implantation in Single-Sided Deafness: A Two-Year Prospective Study
Source: J Clin Med. 2026 Jan 13;15(2):644. doi: 10.3390/jcm15020644 (PMC12842105; doi:10.3390/jcm15020644)
Supplement: Supplementary file 1 [file jcm-15-00644-s001.zip › Supplementary Table S1.pdf]

Supplementary Table S1. Longitudinal changes from baseline to follow-up with effect sizes and 95% confidence intervals.

Values are median [Q1–Q3]. Effect size is Wilcoxon rank-biserial correlation (r_rb) with bootstrap 95% CI (2,000 resamples). Positive r_rb indicates higher follow-up scores relative to baseline; negative r_rb indicates lower follow-up scores. Holm-adjusted p-values are corrected within each outcome across the three baseline-to-follow-up contrasts.

Outcome	Comparison	n (pairs)	n (non-zero diffs)	Baseline median [Q1–Q3]	Follow-up median [Q1–Q3]	Effect size (rank-biserial r)	95% CI (bootstrap)	Wilcoxon p	Holm-adjusted p (within outcome)
FS (Freiburg monosyllables, %; 65 dB)	Baseline vs 6 months	48	47	0 [0–0]	60 [38.75–70]	1.0	[1; 1]	2.35e-09	7.04e-09
FS (Freiburg monosyllables, %; 65 dB)	Baseline vs 1 year	49	46	0 [0–0]	65 [50–75]	1.0	[1; 1]	3.44e-09	7.04e-09
FS (Freiburg monosyllables, %; 65 dB)	Baseline vs 2 years	33	32	0 [0–0]	67.5 [50–75]	1.0	[1; 1]	7.62e-07	7.62e-07
NCIQ 1	Baseline vs 6 months	54	52	70 [57.5–80.62]	73.75 [62.5–83.12]	0.3	[-0; 0.59]	0.063	0.126
NCIQ 1	Baseline vs 1 year	61	56	70 [57.5–80.62]	75 [59–86.5]	0.27	[-0.04; 0.56]	0.078	0.126
NCIQ 1	Baseline vs 2 years	46	45	70 [57.5–80.62]	75 [60.56–87.5]	0.4	[0.08; 0.69]	0.021	0.062
NCIQ 2	Baseline vs 6 months	54	51	72.5 [64.38–85]	77.5 [64.82–87.5]	0.24	[-0.08; 0.54]	0.142	0.427
NCIQ 2	Baseline vs 1 year	61	57	72.5 [64.38–85]	77.78 [65–87.5]	0.16	[-0.14; 0.46]	0.288	0.427

Outcome	Comparison	n (pairs)	n (non-zero diffs)	Baseline median [Q1–Q3]	Follow-up median [Q1–Q3]	Effect size (rank-biserial r)	95% CI (bootstrap)	Wilcoxon p	Holm-adjusted p (within outcome)
NCIQ 2	Baseline vs 2 years	45	43	72.5 [64.38–85]	77.5 [63.12–85]	0.25	[-0.08; 0.56]	0.148	0.427
NCIQ 3	Baseline vs 6 months	54	48	87.5 [67.5–95.56]	86.11 [69.44–92.86]	0.09	[-0.23; 0.4]	0.587	0.823
NCIQ 3	Baseline vs 1 year	61	55	87.5 [67.5–95.56]	86.11 [71.81–95]	0.13	[-0.18; 0.43]	0.411	0.823
NCIQ 3	Baseline vs 2 years	45	42	87.5 [67.5–95.56]	83.33 [72.22–94.69]	0.25	[-0.09; 0.56]	0.156	0.467
NCIQ 4	Baseline vs 6 months	54	51	53.9 [43.92–62.5]	58.33 [43.44–67.99]	0.43	[0.13; 0.69]	0.008	0.008
NCIQ 4	Baseline vs 1 year	61	57	53.9 [43.92–62.5]	57.5 [47.35–70]	0.49	[0.21; 0.72]	0.001	0.003
NCIQ 4	Baseline vs 2 years	46	44	53.9 [43.92–62.5]	57.5 [51.25–72.36]	0.63	[0.38; 0.85]	0.000246	0.000739
NCIQ 5	Baseline vs 6 months	54	52	52.5 [39.73–64.17]	57.5 [46.74–72.5]	0.51	[0.24; 0.74]	0.001	0.001
NCIQ 5	Baseline vs 1 year	61	58	52.5 [39.73–64.17]	58.33 [43.75–81.88]	0.57	[0.32; 0.79]	0.00017	0.000499

Outcome	Comparison	n (pairs)	n (non-zero diffs)	Baseline median [Q1–Q3]	Follow-up median [Q1–Q3]	Effect size (rank-biserial r)	95% CI (bootstrap)	Wilcoxon p	Holm-adjusted p (within outcome)
NCIQ 5	Baseline vs 2 years	46	44	52.5 [39.73–64.17]	60 [45–75]	0.65	[0.39; 0.86]	0.000166	0.000499
NCIQ 6	Baseline vs 6 months	54	51	55.9 [43.75–65.89]	61.11 [50–75.7]	0.58	[0.32; 0.81]	0.000325	0.000325
NCIQ 6	Baseline vs 1 year	61	58	55.9 [43.75–65.89]	67.5 [44.44–78.12]	0.66	[0.43; 0.86]	1.28e-05	3.85e-05
NCIQ 6	Baseline vs 2 years	46	44	55.9 [43.75–65.89]	67.86 [53.89–75]	0.72	[0.47; 0.91]	3.25e-05	6.51e-05
NCIQ total	Baseline vs 6 months	54	53	65.07 [55.78–72.65]	67.57 [59.25–76.85]	0.56	[0.31; 0.78]	0.000354	0.001
NCIQ total	Baseline vs 1 year	61	61	65.07 [55.78–72.65]	69.74 [55.39–82.05]	0.5	[0.24; 0.73]	0.000637	0.001
NCIQ total	Baseline vs 2 years	45	45	65.07 [55.78–72.65]	70.04 [56.4–79.13]	0.59	[0.3; 0.83]	0.000351	0.001
OI directional hearing	Baseline vs 6 months	54	48	2 [1.5–3]	3 [2.38–4]	0.9	[0.77; 0.98]	5.01e-08	1.5e-07
OI directional hearing	Baseline vs 1 year	60	46	2 [1.5–3]	3 [2.5–4]	0.83	[0.64; 0.96]	7.49e-07	1.5e-06
OI directional hearing	Baseline vs 2 years	44	38	2 [1.5–3]	3 [2.5–4]	0.82	[0.61; 0.98]	8.48e-06	8.48e-06

Outcome	Comparison	n (pairs)	n (non-zero diffs)	Baseline median [Q1–Q3]	Follow-up median [Q1–Q3]	Effect size (rank-biserial r)	95% CI (bootstrap)	Wilcoxon p	Holm-adjusted p (within outcome)
OI hearing in silence	Baseline vs 6 months	54	48	3.9 [3.4–4.2]	4.4 [3.8–4.8]	0.57	[0.29; 0.82]	0.000619	0.001
OI hearing in silence	Baseline vs 1 year	60	52	3.9 [3.4–4.2]	4.2 [3.8–4.8]	0.65	[0.41; 0.86]	4.25e-05	0.000127
OI hearing in silence	Baseline vs 2 years	44	38	3.9 [3.4–4.2]	4.4 [3.8–4.6]	0.62	[0.32; 0.87]	0.000859	0.001
OI hearing in noise	Baseline vs 6 months	54	49	2.6 [2.15–3]	3.4 [2.73–3.8]	0.91	[0.79; 0.99]	2.89e-08	5.77e-08
OI hearing in noise	Baseline vs 1 year	60	53	2.6 [2.15–3]	3.4 [2.8–4]	0.9	[0.78; 0.98]	1.27e-08	3.81e-08
OI hearing in noise	Baseline vs 2 years	44	42	2.6 [2.15–3]	3.4 [2.6–3.8]	0.8	[0.58; 0.97]	5.75e-06	5.75e-06
OI Total	Baseline vs 6 months	54	54	3.04 [2.67–3.38]	3.7 [3.15–4.11]	0.84	[0.68; 0.96]	7.56e-08	1.51e-07
OI Total	Baseline vs 1 year	60	58	3.04 [2.67–3.38]	3.75 [3.11–4.08]	0.85	[0.71; 0.95]	1.85e-08	5.56e-08
OI Total	Baseline vs 2 years	44	44	3.04 [2.67–3.38]	3.75 [3.17–4.17]	0.82	[0.6; 0.97]	2.5e-07	2.5e-07
TQ Total	Baseline vs 6 months	52	50	24 [11–51]	9.5 [1.75–28]	-0.85	[-0.97; -0.68]	1.56e-07	4.68e-07
TQ Total	Baseline vs 1 year	56	52	24 [11–51]	10 [0.25–36.5]	-0.76	[-0.92; -0.54]	1.69e-06	3.38e-06
TQ Total	Baseline vs 2 years	47	44	24 [11–51]	11.5 [3.75–30.75]	-0.61	[-0.84; -0.34]	0.000386	0.000386
PSQ	Baseline vs 6 months	54	51	0.35 [0.23–0.52]	0.29 [0.18–0.47]	-0.57	[-0.8; -0.3]	0.000395	0.001

Outcome	Comparison	n (pairs)	n (non-zero diffs)	Baseline median [Q1–Q3]	Follow-up median [Q1–Q3]	Effect size (rank-biserial r)	95% CI (bootstrap)	Wilcoxon p	Holm-adjusted p (within outcome)
PSQ	Baseline vs 1 year	60	56	0.35 [0.23–0.52]	0.28 [0.15–0.45]	-0.38	[-0.62; -0.09]	0.014	0.028
PSQ	Baseline vs 2 years	47	45	0.35 [0.23–0.52]	0.33 [0.19–0.47]	-0.36	[-0.65; -0.04]	0.037	0.037
ADS-L	Baseline vs 6 months	55	51	12 [7–23]	11 [5–20]	-0.27	[-0.55; 0.05]	0.091	0.273
ADS-L	Baseline vs 1 year	60	56	12 [7–23]	12.5 [6–20.75]	-0.15	[-0.43; 0.16]	0.339	0.532
ADS-L	Baseline vs 2 years	45	43	12 [7–23]	11 [6–22]	-0.19	[-0.53; 0.12]	0.266	0.532
GAD-7	Baseline vs 6 months	55	43	4 [2–8]	4 [1–7]	-0.38	[-0.68; -0.05]	0.028	0.085
GAD-7	Baseline vs 1 year	60	48	4 [2–8]	4 [1–6]	-0.28	[-0.57; 0.02]	0.087	0.174
GAD-7	Baseline vs 2 years	44	39	4 [2–8]	5 [2–7]	-0.13	[-0.48; 0.23]	0.479	0.479
