# Supplementary material for: Long-Term Auditory, Tinnitus, and Psychological Outcomes After Cochlear Implantation in Single-Sided Deafness: A Two-Year Prospective Study
Source: J Clin Med. 2026 Jan 13;15(2):644. doi: 10.3390/jcm15020644 (PMC12842105; doi:10.3390/jcm15020644)
Supplement: Supplementary file 1 [file jcm-15-00644-s001.zip › Supplementary Table S2.pdf]

**Supplementary Table S2. Attrition analysis:** baseline characteristics of participants with versus without complete 2-year core outcome data.

Completers were defined as participants with non-missing 2-year data for all core outcomes (Freiburg monosyllables, OI Total, NCIQ total, TQ Total, and PSQ total). Continuous variables are median [Q1–Q3] and compared using Mann–Whitney U tests. Categorical variables are n (%) and were compared between completers and non-completers using two-sided Fisher's exact tests (2×2).

| Variable | Completers (2-year) (n=29) | Non-completers (n=41) | p-value |
|---|---|---|---|
| Age at surgery (years) | 61 [52–71] | 60.5 [47–69.25] | 0.67 |
| Duration of tinnitus (years) | 10 [9–11] | 10 [10–11.5] | 0.332 |
| Freiburg monosyllables, poorer ear (%, 65 dB) | 0 [0–0] | 0 [0–0] | 0.276 |
| OI Total (baseline) | 3 [2.75–3.33] | 3.08 [2.67–3.52] | 0.909 |
| NCIQ total (baseline) | 63.64 [55.1–71.98] | 67.8 [56.52–72.8] | 0.731 |
| TQ Total (baseline) | 25 [9–38] | 24 [11–54.5] | 0.479 |
| PSQ total (baseline) | 0.37 [0.28–0.51] | 0.32 [0.18–0.52] | 0.328 |
| ADS-L (baseline) | 12 [9–21] | 10 [6–24.5] | 0.887 |
| GAD-7 (baseline) | 4 [2–7] | 5 [2–8.75] | 0.698 |
| Sex | | | 0.809 |
| male | 11 (37.9%) | 17 (41.5%) | |
| female | 18 (62.1%) | 24 (58.5%) | |
| Missing | 0 (0.0%) | 0 (0.0%) | |
| Presence of tinnitus | | | 0.297 |
| yes | 12 (41.4%) | 10 (24.4%) | |
| no | 4 (13.8%) | 8 (19.5%) | |
| Missing | 13 (44.8%) | 23 (56.1%) | |
